# Redes sociales, adolescencia y trastornos de la conducta alimentaria: la necesidad de una mirada comprehensiva

**DOI:** 10.23938/ASSN.1075

**Published:** 2024-04-17

**Authors:** Charo Sádaba

**Affiliations:** 1 Universidad de Navarra Facultad de Comunicación Pamplona España; 2 Universidad de Navarra Instituto Cultura y Sociedad Pamplona España

La preocupación por la salud mental de la población, y en especial de los más jóvenes, crece a nivel global, particularmente tras la pandemia de COVID-19. De acuerdo con el IV Barómetro Juvenil, en España un 59,3% de jóvenes entre 15 y 29 años manifestaba en 2021 haber tenido algún problema de salud mental, dato que supone un aumento significativo desde el 28,4% que arrojaba el mismo indicador en 2017^1^. En este mismo estudio, el 6,1% de los jóvenes declara, además, tener un diagnóstico de trastorno de conducta alimentaria (TCA).

En este contexto de incertidumbre por la salud mental de este grupo de edad, el papel que puede jugar la tecnología, y en particular las redes sociales, está sometido a un juicio creciente por parte de la opinión pública, las autoridades y también por la investigación. Concretamente, preocupa el hecho de que los adolescentes estén expuestos a una constante corriente de imágenes y mensajes que promueven estándares de belleza irreales por el posible impacto que puede tener en sus hábitos alimenticios y de ejercicio físico y, en último término, sobre posibles patologías.

A nadie escapa la importancia del culto al cuerpo en las sociedades occidentales avanzadas que, pese a los esfuerzos constantes a favor de la diversidad corporal, como el *body positive*, no dejan de señalar determinados cánones de belleza como normativos y, por tanto, deseables. Aunque la batalla alrededor del cuerpo femenino ideal parece tener más impacto en las niñas y adolescentes, también el cuerpo masculino se ha convertido en objeto de discusión y de anhelo para muchos adolescentes y jóvenes^2^. Las redes sociales son el escenario preferente de esta narrativa, donde se desarrolla con más fluidez e intensidad y donde con más facilidad alcanza a los públicos menores de edad.

La vinculación de esta exposición constante a imágenes idealizadas, junto con la enorme disponibilidad de servicios, productos y consejos para lograr un cuerpo perfecto, bien a través de la alimentación o del ejercicio^3^, hace plantearse cómo de protegidos están los más jóvenes ante estos discursos que pueden tener un impacto negativo sobre su salud mental y física.

## LA INFLUENCIA DE LAS REDES SOCIALES

Es evidente que los contenidos a los que este grupo de edad accede a través de internet pueden influir en la configuración de los estándares de lo que es socialmente aceptable, deseable o bueno, especialmente porque su estado de desarrollo madurativo los convierte en público vulnerable. Las redes sociales, a diferencia de los medios tradicionales, se adaptan a los intereses de sus usuarios y animan a su interacción constante, logrando un entorno más inmersivo y potencialmente más influyente^4^. Son, además, espacios sociales y de ocio, donde el pensamiento crítico ante los contenidos se reduce al tiempo que se incrementa el tiempo invertido. Es también innegable la accesibilidad a esos servicios y contenidos gracias a la generalización del teléfono móvil con acceso a internet en prácticamente todas las franjas de edad a partir de los 11 años.

Alguna de estas redes sociales, como Instagram o TikTok, son espejos en los que las personas usuarias buscan referentes, tendencias o modelos en los que inspirarse o a quienes imitar en sus estilos de vida. Esta búsqueda de información hace posible, a través de los algoritmos, que estos mismos usuarios reciban una publicidad muy personalizada que se adapta a aquello que están buscando. Y a estos contenidos publicitarios explícitos se añade que muchos de los modelos a quienes siguen de manera voluntaria persiguen a su vez intereses comerciales, ya que están patrocinados por marcas o bien buscan monetizar su presencia en redes sociales logrando audiencias masivas y contenidos virales.

Destaca aquí el papel de los creadores de contenidos, conocidos como *influencers*, que llevan a cabo de manera muy eficiente estrategias para captar la atención y la lealtad de sus seguidores^5^. Lo que podría ser un caso de abuso de confianza particularmente doloso cuando incumbe a menores de edad con una capacidad crítica en desarrollo, adquiere un tinte diferente al tratar sobre cuestiones que pueden impactar directamente sobre su salud mental y física^3^^,^^6^. La propia naturaleza visual y estilística de plataformas como Instagram puede constituir un riesgo, particularmente acentuado entre las chicas^7^ y aquellos que muestran un uso problemático de redes sociales^8^.

## REDES SOCIALES, ADOLESCENTES Y TRASTORNOS DE CONDUCTA ALIMENTARIA

Ya antes de la popularización de las redes sociales, internet se había convertido en un espacio donde quienes sufrían un TCA podían encontrar consejos, ideas o apoyo. Gina Lladó y col^9^ analizaron cómo estos contenidos encontraron su acomodo en blogs, grupos de Facebook u otras vías digitales. No obstante, el problema se magnifica con la llegada de las redes sociales: no se trata tan solo de que quienes sufren, o son particularmente propensos a sufrir un TCA tengan acceso a redes de apoyo y a consejos que les ayuden a disimularlo, sino que la propia cultura visual y centrada en la exaltación de la apariencia física contribuye a consolidar determinados modelos de cuerpo ideal, que llegan de manera más personalizada a públicos más amplios. Cómo esta exposición puede afectar y acelerar la aparición de relaciones poco saludables con la alimentación o terminar en un TCA ha sido objeto de atención de los académicos en los últimos años.

Un estudio de Beatriz Feijoo y col publicado en 2023 ponía de manifiesto la influencia que las redes sociales ejercían sobre la percepción del aspecto físico de los adolescentes españoles, y también cómo los *influencers* eran, en muchos casos, fuente de pautas y sugerencias sobre alimentos, pautas de alimentación y de ejercicio físico para lograr cuerpos ideales^2^. Estos contenidos, consumidos a través de las redes sociales, generan un posible círculo vicioso en el que los adolescentes replican lo que ven hacer a los creadores de contenido y lo comparten con sus amigos y conocidos. El posible impacto negativo se incrementa cuando estas publicaciones pueden generar una respuesta en forma de “*me gusta*” o de comentario positivo, lo que refuerza su determinación.

Esto indica que la presión social propia de la etapa adolescente, cuando el deseo de sentirse aceptado por el grupo de referencia es muy radical, se extiende también a las redes. Esta presión del grupo puede tener un efecto de refuerzo positivo, que impulse a realizar una acción saludable o entretenida, por ejemplo, pero sin duda también negativo como cuando un comentario genera un sentimiento negativo de tristeza o rechazo. En cualquier caso, es necesario tener en cuenta este factor cuando se estudia y se trabaja con esta franja de edad.

Hay acuerdo en que la relación entre consumo de redes sociales, preocupación por la imagen corporal y trastornos alimenticios es significativa pero compleja. Así lo muestra la *scoping review* más reciente, publicada en 2023 por Dane y Bathia^10^, pero también la revisión de Lozano-Muñoz y col realizada en 2022 concluyó que “*a pesar de algunos aspectos positivos, las RRSS promueven cánones de belleza basados en la delgadez, permiten la comparación entre iguales incrementando la preocupación por el peso, y crean espacios que fomentan los trastornos de la conducta alimentaria*”^7^.

Pero pese a estas evidencias, que no dejan de ser todavía parciales y limitadas, Dane y Bathia concluyen diciendo que “*mientras que nuestra revisión señala que puede haber implicaciones de gran escala entre los casi 4.000 millones de usuarios de redes sociales en todo el mundo, es importante también hacer notar que no todos los usuarios tienen una mala percepción de su propio cuerpo o desarrollan un TCA. Esto hace que haya que preguntarse ¿qué es lo que hace a ciertos individuos más susceptibles*?”^10^.

## MIRANDO AL FUTURO

Todas las investigaciones concuerdan en la necesidad de reforzar cualquier afirmación sobre el vínculo entre redes sociales y TCA con más investigación^8^. La influencia del género^7^, de la edad^11^ o de un uso problemático de las redes sociales^8^ ya han sido analizadas y convendría replicar estudios en otras áreas geográficas y culturales para poder consolidar los resultados obtenidos. En cualquier caso, establecer una relación única y de causalidad entre redes sociales y TCA parece poco oportuna y poco pertinente.

Para Marks y col^4^ (2020), esta ola creciente que vincula imagen corporal y TCA en las redes sociales debería conllevar también un cambio en las estrategias de promoción de la salud. La literatura, según estos autores, confirma que los enfoques de salud centrados en el peso tienen un impacto negativo sobre la salud física y el bienestar psicológico. En muchas ocasiones son profesionales de la salud quienes, como creadores de contenidos en las redes sociales, divulgan consejos para una vida saludable. Convendría también analizar cuáles son sus argumentos y el papel que el peso corporal tiene en ellos.

Esto podría conllevar a la reflexión de los responsables de los sistemas de salud sobre en qué medida disponen de estrategias dirigidas a ofrecer respuestas a las preguntas que se puede hacer este grupo de edad, o para salir al paso, de manera efectiva, de los problemas que suscita. Probablemente esto implique también cambiar algunas pautas en las consultas de atención primaria, incluyendo preguntas y consejos sobre el papel de la tecnología que puedan ayudar a los jóvenes, y también a sus familias.

En el presente número de *Anales del Sistema Sanitario de Navarra* se publica el artículo de revisión *Efectividad de las intervenciones para mitigar la influencia de las redes sociales en la anorexia y la bulimia. Una revisión sistemática*, cuyos autores son Noelia Lozano-Muñoz, Álvaro Borrallo-Riesgo y Maria Dolores Guerra-Martín^12^. En él se apunta a la importancia de las “*intervenciones de carácter educativo centradas en prevención y promoción de la salud, autocrítica, autopercepción, autoestima, imagen corporal, manejo nutricional y alfabetización digital*” como vías a través de las que limitar el impacto negativo que las redes sociales pueden tener sobre los trastornos de conducta alimentaria. Parece particularmente relevante esta aportación porque, además de reclamar más investigación, también anima a una visión interdisciplinar de este problema complejo que dista mucho de tener una solución simple.

Quizá este enfoque interdisciplinar puede ayudar a enriquecer estos resultados incipientes y a ofrecer nuevas alternativas que ayuden a frenar el auge de estos problemas entre un grupo de edad particularmente vulnerable.
